# QT Prolongation and Associated Ventricular Tachycardia due to Cardiac Iron Load in a Patient with Thalassemia Major

**DOI:** 10.1155/2019/5791094

**Published:** 2019-06-17

**Authors:** Derya Demirtas, Abdullah Orhan Demirtas, Hilmi Erdem Sumbul, Ayse Selcan Koc

**Affiliations:** ^1^Assistant Professor in Department of Internal Medicine, Adana Health Practice and Research Center, Health Sciences University, Adana, Turkey; ^2^Assistant Professor in Department of Cardiology, Adana Health Practice and Research Center, Health Sciences University, Adana, Turkey; ^3^Assistant Professor in Department of Radiology, Adana Health Practice and Research Center, Health Sciences University, Adana, Turkey

## Abstract

We report the case of a 23-year-old male with thalassemia major who developed long QT and continuous ventricular tachycardia (VT). Electrocardiography, echocardiography, and cardiac magnetic resonance imaging (MRI) were used for diagnosis and risk stratification. VT causes and treatments are presented and discussed. Ventricular arrhythmia can be treated by normalizing QT interval with high-dose beta-blocker therapy. However, MRI-compatible internal cardiac defibrillator implantation was performed due to the high risk in this patient.

## 1. Introduction

Excess iron loading occurs in many organs, primarily the heart and liver, and in patients with thalassemia major. In a study using magnetic resonance imaging (MRI) on a large number of patients, serious heart involvement was reported to be 20%, while in 58% of patients, iron deposition was reported to be within normal limits [1]. Malignant ventricular arrhythmias, which can result in sudden cardiac death due to increased cardiac repolarization, can be seen in patients who develop heart failure due to iron deposition [2]. In the following article, we discussed the treatment of the patient with thalassemia major who developed long QT and continuous ventricular tachycardia (VT) due to severe iron deposition.

## 2. Clinical Case

**Figure 1:**
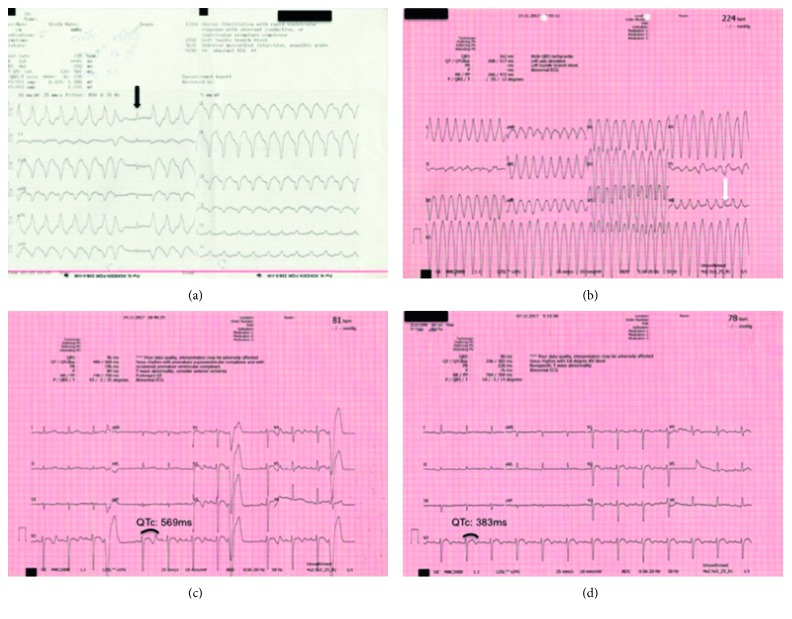
Electrocardiographies (ECGs) of patients. (a) Ventricular tachycardia (VT) ECG in emergency department; the capture beat, which is a diagnostic ECG finding for VT, is indicated by the arrow. (b) Another VT ECG in cardiac intensive care unit, another diagnostic ECG finding for VT, and the fusion beat is shown with an arrow. (c) Sinus rhythm ECG after cardioversion (ventricular premature beats (R on T) and long QT/QTc). (d) QT-QTc intervals were corrected and ventricular premature beats disappeared with maximum tolerable doses of beta-blocker therapy.

A 23-year-old male with a previous diagnosis of beta thalassemia major was admitted to the emergency department with complaints about palpitation, dizziness, blurred vision, weakness, and tiredness. His electrocardiography (ECG) showed VT (Figure 1(a)); thus, the patient was taken to the cardiac intensive care unit (CICU). Serum electrolytes were normal in the emergency department (Na: 138 mmol/lt, K: 4.2 mmol/lt, Ca: 9.8 mg/dl, and Mg: 2.4 mg/dl). The VT continued when the patient was admitted to the CICU (Figure 1(b)). Blood pressure was 80/60 mmHg. He had rough breathing. Synchronized, 100 J biphasic cardioversion was performed by sedating the patient who had an ECG compatible with VT under emergency conditions. VT was successfully corrected with cardioversion. The ECG was in the sinus rhythm and had ventricular premature beats (R on T) and QT-QTc interval prolongations (Figure 1(c)). Immediate beta-blocker treatment was initiated. On the 13^th^ day of treatment, QT-QTc intervals were corrected and ventricular premature beats disappeared with maximum tolerable doses of beta-blocker therapy (Figure 1(d)). He was taking deferoxamine methanesulfonate 500 mg daily for blood chelation. Hemoglobin value was measured as 8.6 mg/dl in laboratory findings. A unit of blood transfusion was given with the recommendation of the Hematology Clinic. Hemoglobin value after transfusion was 9.6 gr/dl, serum ferritin was >1500 ng/l, and serum iron was 251 pg/dl.

**Figure 2:**
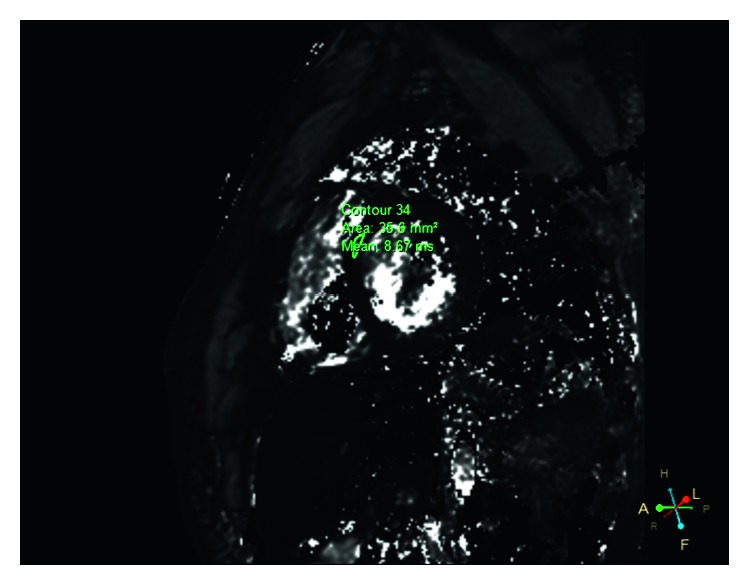
Cardiac magnetic resonance imaging (MRI) finding: T2^*∗*^ mapping MRI sagittal midventricular view shows a dark appearance of left ventricle myocardium due to severe iron load (T2^*∗*^ value was 8.67 ms).

Echocardiography revealed that ejection fraction was 69%, interventricular septum was hypertrophic (1.5 cm), left atrium was dilated (end-diastolic diameter 4.1 cm), and stage III diastolic dysfunction (restrictive filling pattern) was observed. A cardiac MRI was requested to screen the iron deposition in the heart of the patient. On MRI imaging, left ventricle (LV) was diffuse hypertrophic and hypointense (dark) (myocardial T2^*∗*^ value 8.67 milliseconds). However, right ventricle (RV) myocardium was normal, stained with MRI (Figure 2). MRI results were interpreted as deposition of intense iron in the LV myocardium (Figure 2). As a result of the examinations, it was thought that VT might be related to QT prolongation related to iron deposition in the heart. Electrophysiology study was performed when the patient had normal QT interval and tachycardia was not induced in the procedure. Current situation was told to the patient and relatives, secondary internal cardiac defibrillator (ICD) implantation was decided for prophylaxis, and Medtronic brand MRI compatible DDD-ICD implantation was performed. Beta-blocker treatment continued. The patient did not have any VT recordings at routine polyclinic and ICD control after a month.

## 3. Discussion

In thalassemia major, due to ineffective erythropoiesis, regular blood transfusions are needed and this may lead to excess iron deposition in the liver and heart. These patients are lost due to cardiac failure and cardiac complications that occur as a result of iron deposition in the heart. In patients with thalassemia major, mortality rates reduced significantly due to intravenous and oral chelation therapy [3, 4]. Our patient could have reached the age of 23 because he had received regular chelation therapy.

In patients with thalassemia major, MRI imaging is widely used to detect early onset and general iron overload [1]. In addition, information about RV and LV anatomy and functions can be obtained with MRI. In cardiac MRI, patients with iron loads of T2^*∗*^ <10 milliseconds were considered serious and those with T2^*∗*^ >20 milliseconds were considered normal [5]. In studies conducted, 83% of patients with cardiac arrhythmias and iron-overload-induced cardiomyopathy had MRI T2^*∗*^ levels <20 milliseconds [5]. Our patient also had an iron load of T2^*∗*^ = 8.6 milliseconds and was evaluated in the group with severe iron overload.

In thalassemia major, due to iron overload and cardiac fibrosis, fatal arrhythmias such as continuous VT and ventricular fibrillation may occur [2]. In various studies, parameters such as QT, QTc, and QTp in superficial ECG have been shown to increase significantly in comparison with normal healthy subjects [6]. In our case, QT interval was 490 milliseconds and QTc interval was 569 milliseconds. In our patient, there was also premature ventricular (R on T) beats. Beta-blocker therapy normalized both QT and QTc intervals, and ventricular premature beats disappeared. This suggests that beta-blocker treatment may routinely be initiated in patients with thalassemia major who have ventricular arrhythmia, if not contraindicated.

Because the current guideline [7] considers it as a reversible condition with chelation therapy, it recommends chelation and drug treatment in primer protection and does not recommend ICD in both primary and secondary protection. Information on antiarrhythmic therapy is limited in patients with thalassemia major and accompanying ventricular arrhythmia. The DDD-ICD device was implanted prophylactically on his own request because of the young age of the patient, history of having VT twice, his undergoing iron chelation therapy, and increased risk of sudden cardiac death. Personalized treatment is usually applied in patients with VT due to iron overload. In literature, ICD implantation was performed in two similar cases [4, 8]. In our case, the implantation of DDD-ICD was coordinated with the decision of 3 electrophysiologists.

In conclusion, in patients with thalassemia major, long QT and subsequent VT may occur due to excess iron accumulation. In these patients, ventricular arrhythmia can be treated by normalizing the QT interval with high-dose beta-blocker therapy after acute condition treatment. Beta-blocker therapy must be considered in thalassemia major patients with excess iron deposition and associated ventricular arrhythmia, and ICD therapy should be considered in patients at high risk.
